# Pathogenic *APC* Variants in Latvian Familial Adenomatous Polyposis Patients

**DOI:** 10.3390/medicina55100612

**Published:** 2019-09-20

**Authors:** Zanda Daneberga, Dace Berzina, Viktors Borosenko, Zita Krumina, Linda Kokaine-Sapovalova, Andris Gardovskis, Egija Berga-Svitina, Janis Gardovskis, Edvins Miklasevics

**Affiliations:** 1 Institute of Oncology, Riga Stradiņš University, LV-1007 Riga, Latvia; 2 Department of Surgery, Pauls Stradiņš Clinical University Hospital, LV-1002 Riga, Latvia; 3 Department of Biology and Microbiology, Riga Stradiņš University, LV-1007 Riga, Latvia; 4 Department of Surgery, Riga Stradiņš University, LV-1007, Riga, Latvia

**Keywords:** FAP, *APC* gene, CRC, pathogenic variants, germline variants

## Abstract

*Background and objectives:* Familial adenomatous polyposis is one of the *APC*-associated polyposis conditions described as genetically predetermined colorectal polyposis syndrome with a variety of symptoms. The purpose of this study was to determine sequence variants of the *APC* gene in patients with familial adenomatous polyposis (FAP) phenotype and positive or negative family history. *Materials and Methods:* Eight families with defined criteria of adenomatous polyposis underwent molecular genetic testing. Coding regions and flanking intron regions of the *APC* gene were analyzed by Sanger sequencing. *Results:* Eight allelic variants of the *APC* gene coding sequence were detected. All allelic variants of the *APC* gene were predicted to be pathogenic based on criteria according to the “Joint Consensus Recommendation of the American College of Medical Genetics and Genomics and the Association for Molecular Pathology” (2015), four of them c.1586_1587insAT, c.2336delT, c.3066_3067insGA, and c.4303_4304insC, were considered novel. *Conclusions:* The timely molecular genetic analysis of *APC* germline variants and standardized interpretation of the pathogenicity of novel allelic variants has a high impact on choice for treatment, cancer prevention, and family genetic counseling.

## 1. Introduction

*APC*-associated polyposis conditions include three main clinical phenotypes of polyposis: familial adenomatous polyposis (FAP), attenuated FAP (AFAP), and gastric adenocarcinoma and proximal polyposis of the stomach (GAPPS) [1]. The Online Mendelian Inheritance in Man (OMIM) database in phenotype familial adenomatous polyposis (FAP1, # 175100) also includes Gardner syndrome (GS) and brain tumor-polyposis syndrome 2 (BTPS2) [2]. All these clinical phenotypes are caused by heterozygous pathogenic variants of the *APC* gene (APC regulator of Wnt signaling pathway), encoding a multifunctional gatekeeper tumor suppressor protein with ubiquitous expression in brain, colon and 23 other tissues [1,3,4].

Familial adenomatous polyposis (FAP) is a genetically predetermined colorectal polyposis syndrome with a variety of symptoms. Approximately 1% of colorectal cancer (CRC) cases are due to FAP [5]. The reported incidence of FAP among CRC patients in Latvia is 0.08% [6]. The main findings in FAP patients are hundreds to thousands of polyps and adenomas in the colorectum as well as extra-colonic findings, such as desmoid tumors, osteomas, epidermoid cysts, etc. The development of adenomas can be seen in late childhood and early adolescence. Most FAP patients (almost 100%, if left untreated) develop colorectal cancer at a mean age of 40 to 50 years. In the case of AFAP, the development of polyps starts later (between the age of 40 and 70), and fewer polyps are present (around 30). The development of CRC in AFAP patients is estimated to be 70% by age 80 [3,5].

FAP is caused by germline variants of the *APC* gene in about 80% to 95% of patients. In this case, the phenotype is considered to be associated with *APC*-associated polyposis conditions. The inheritance of FAP is autosomal dominant. Approximately 20% to 25% of allelic variants of the *APC* gene arise de novo; the rest are inherited [1]. Various online databases and scientific articles widely describe allelic variants in the gene. Based on information from the Leiden Open Variation Database (LOVD) database, 1692 unique allelic variants of the *APC* gene are included in the database (information retrieved on 16/06/2019) [7]. The rest of the FAP phenotype is caused by biallelic variants in the *MUTYH* gene. The clinical phenotype of *MUTYH*-associated polyposis (MAP) is similar to AFAP. MAP has an autosomal recessive type of inheritance [8]. In this article, only FAP cases with allelic variants of the *APC* gene will be considered. This article concerns only *APC* germline variants associated with mainly classical FAP phenotype.

*APC* is a tumor-suppressor gene with a central role in the Wnt signaling pathway. The gene is located on chromosome 5 locus q21-q22 and consists of 18 exons (LRG_130t1; NM_000038.4). The gene has three transcripts and encodes for a 2843 amino acid long polypeptide [4,5,9]. The APC protein (UniProtKB-P25054) acts as an antagonist in the Wnt signaling pathway and is also involved in processes of cell migration and adhesion, activation of transcription, and apoptosis [4,10]. Around 95% of germline variants of the *APC* gene are nonsense or frameshift variants leading to a truncated protein. Most of the germline variants occurring in the 5’ coding half of the gene cause loss of the β-catenin level regulating domain, axin binding domain as well as C-terminus microtubule and EB1 binding domains [11].

The main goal of this study was to summarize current findings of the *APC* germline variants in Latvian FAP patients, report novel variants, and evaluate their pathogenicity.

## 2. Materials and Methods

### 2.1. Patients

Patients corresponding to defined criteria of adenomatous polyposis were sent to Riga Stradins University Institute of Oncology for molecular genetics analysis of the *APC* gene. Eight unrelated patients were involved from Pauls Stradins Clinical University Hospital, Liepaja Regional Hospital, and University Children’s Hospital. After the FAP diagnosis was confirmed, the family genetic consultation and cascade testing were performed. None of the patients’ family members had previously been screened for APC mutations. The genetic study was performed with the approval of the Central Committee of Medical Ethics (decision No1/26-05-17 of 26 January 2017) and written informed consent was obtained from all involved patients.

### 2.2. Methods

DNA was extracted from whole blood using the QIAgen FlexiGene DNA Kit (Qiagen, Hilden, Germany). All DNA samples were subjected to the Sanger sequencing of coding regions and flanking intron regions of the *APC* gene as described earlier [12] and according to the manufacturer’s protocol (Life Technologies, Carlsbad, CA, USA). Allelic variants were confirmed by sequencing both DNA strands on an independent PCR product. Reactions were analyzed using the Applied Biosystems genetic analyzer ABI3130 (Life Technologies, Carlsbad, CA, USA). Large deletions or duplications of the *APC* gene were not analyzed. For the annotation of APC variants the following databases were used: INSIGHT-group database (http://www.insight-group.org/mutations/), National Center for Biotechnology Information (NCBI) SNP database, LOVD database, The Universal Mutation Database (UMD-APC), NCBI ClinVar database, and *in-silico* prediction tools MutationTaster and Mutalyzer [7,13,14,15,16]. Interpretation of the germline variants was made according to the “Joint Consensus Recommendation of the American College of Medical Genetics and Genomics and the Association for Molecular Pathology” (2015) [17].

## 3. Results

**Figure 1:**
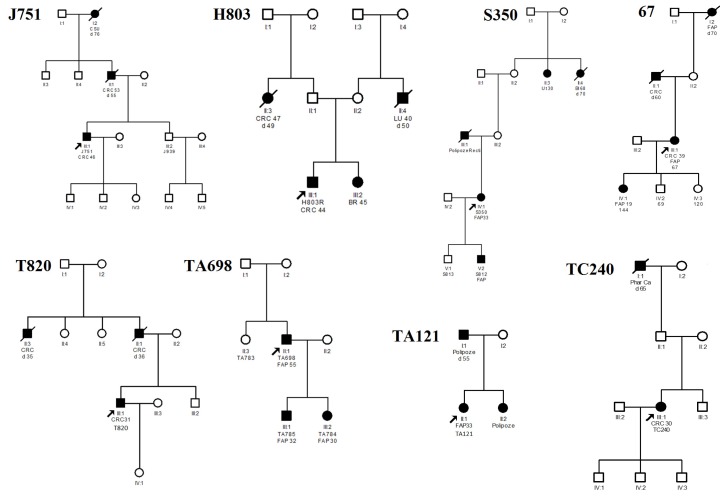
Medical history of cancers in the patients’ families. Abbreviations: CRC—colorectal cancer; Ut—cancer; C Su—cancer site unknown; Li – liver cancer; BR – breast cancer; Phar – pharyngeal cancer; Bl – bladder cancer; d – died.

Genetic analysis of the *APC* gene was requested for eight patients. All eight had positive clinical findings confirming polyposis, and all but one patient had a positive family history of colorectal polyposis or colorectal cancer, according to diagnostic criteria for FAP. The patient family histories of cancer are summarized in Figure 1. Five had carpeted polyposis throughout the colon and rectum, and one had diffuse polyposis of the rectum, stomach, and duodenum. Six out of eight patients had developed colorectal cancer, two of them diagnosed only during total proctocolectomy, with no previous suspicion of this diagnosis, and two patients were without a clinical cancer diagnosis. Two of the patients underwent total proctocolectomy, one patient had left-side colon resection, and one patient has not had colorectal surgery. No information about surgical management was available for three patients. The age range at diagnosis was from 30 to 55 years, with the average age of patient age being 38.88 years (± 8.84). Based on the number of polyps and the age of onset, two of the patients (J751 and TA698) corresponded to the AFAP phenotype. Despite phenotype similarities with AFAP, patient J751 developed an early manifestation of CRC.

**Figure 2:**
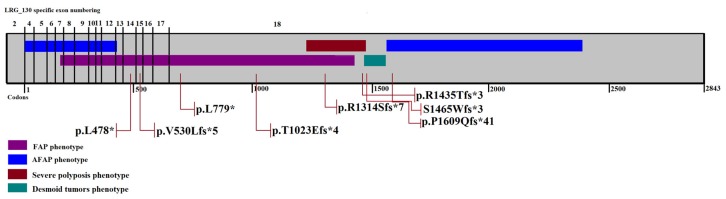
*APC* germline variants identified by exon and codon location. The color bars represent genotype–phenotype correlation described earlier [19,20].

DNA sequencing revealed *APC* germline variants in all eight patients. The identified germline variants and clinical data of patients are summarized in Table 1. All allelic variants were classified as pathogenic, as results in a truncated protein. One of the variants is nonsense and all other frameshifts, leading to shortening of the polypeptide by half or more amino acids. The location of variants according to the exons and codons of the *APC* gene is depicted in Figure 2.

Allelic variant c.1433T>G is described in the ClinVar database as likely pathogenic, but no further information about the clinical background of the patient is available either in databases or the literature. Germline allelic variant c.3942delG is not found in databases but has been described previously in the Latvian population [6]. There is no information available about the known link between families harboring the same allelic variant. Variant c.4826delC is mentioned in the UMD-APC database. Variant c.4393_4394delAG is listed in the NCBI dbSNP with the database number rs387906234. The other four *APC* variants are novel, to the best of our knowledge, previously undescribed. The pathogenicity of novel germline variants was evaluated based on standard recommendations for interpretation of variants observed in patients with suspected inherited disorders or conditions for a clinical diagnostic purpose [17]. The combined criteria for supporting pathogenicity are summarized in Table 2. Genotype–phenotype evaluation showed the consistency of clinical phenotype with the position of allelic variant described earlier, for seven out of eight patients. One phenotype showed overlapping of FAP and AFAP (J751).

Family cascade testing was done in five families. Genetic analysis of *APC* variants was done in children under 18, in two of the families. In one family, with allelic variant c.3066insGA, one of the children was negative for allelic variant in *APC*, while the other was positive and colonoscopy was positive for polyposis in the rectum. In a family with allelic variant c.3942delG, one child out of three was negative for *APC* variant, but two were positive. The eldest daughter at age 18 had polyposis of the rectum. For the youngest daughter, no clinical investigations were done. All other patients from families available for family cascade genetic testing were adults, but no clinical investigation results are available after the results of genetic testing.

## 4. Discussion

The reported incidence of genetically confirmed FAP among CRC patients in Latvia is far lower than that reported worldwide. Most of our reported cases were clinically recognized and requested for molecular genetic testing by surgeons. Only one case was requested by a clinical geneticist. This situation is not unusual and is limited by the age of onset and specific phenotype.

The spectrum of the *APC* gene germline variants in European descent populations is reported extensively [21,22,23,24]. The reported mutational hotspots of the *APC* gene are located in the 5′ part at codons 1309 and 1061, accounting for approximately 17% and 11% of all described germline *APC* variants, respectively. Due to the accumulation of variants from codon 1250 to 1464, this region is termed the mutation cluster region (MCR) [9]. In our study, we did not find any of the two most common germline variants; however, our study group was rather small. Only three out of eight *APC* variants reported by our group are in the MCR.

The population-specific variants are known to be reported from the population with Ashkenazi Jewish ancestry, with two missense variants, which do not lead to polyposis but may develop CRC with a lifetime risk of around 20% [9]. All revealed *APC* variants detected in our study were unique for unrelated patients; however, the size of the study group should be taken into account here. Acquired results demonstrate high heterogeneity of *APC* variants in Latvian FAP patients.

Interpretation of the germline variants was made according to the «Joint Consensus Recommendation of the American College of Medical Genetics and Genomics and the Association for Molecular Pathology» (2015) [17]. This is the most appropriate standardized up-to-date approach for interpretation of sequence variants for clinical use. No functional studies were performed within the scope of this evaluation of reported novel sequence variants, but all available molecular biology data and clinical data were taken into account.

The genotype impact on phenotype is studied by many research groups, leading to the correlation of mutated codons with a clinical phenotype of *APC*-associated polyposis conditions. The detected genotype–phenotype correlation plays an important role in clinical decisions for patient treatment strategy. The clinical phenotype of our patients was consistent with the genotype–phenotype correlation described earlier (Figure 2).

## 5. Conclusions

The study of clinically selected patients revealed eight pathogenic variants of the *APC* gene, four of them—c.1586_1587insAT, c.2336delT, c.3066_3067insGA and c.4303_4304insC, are considered novel. The timely molecular genetic analysis of germline variants and standardized interpretation of the pathogenicity of novel *APC* variants has a high impact on choice for treatment, cancer prevention, and family genetic counseling.

## Figures and Tables

**Table 1:** Identified germline variants and clinical data of probands.

Family (Proband)	Nucleotide Change ^1^	AA Change	Type of Variant	Age of Diagnosis	Polyposis	CRC	Ref.
J751	c.1433T>G	p.L478*	Nonsense	46	<100	+	[14]
H803	c.1586_1587insAT	p.V530Lfs*5	Frameshift	44	Carpeted polyposis	+	novel
TA121	c.2336delT	p.L779*	Frameshift	33	Carpeted polyposis	-	novel
S350	c.3066_3067insGA	p.T1023Efs*4	Frameshift	33	Carpeted polyposis	+	novel
67	c.3942delG	p.R1314Sfs*7	Frameshift	39	Carpeted polyposis	+	[6]
T820	c.4303_4304insC	p.R1435Tfs*3	Frameshift	31	Carpeted polyposis	+	novel
TC240	c.4393_4394delAG	S1465Wfs*3	Frameshift	30	Diffuse polyposis	+	[13]
TA698	c.4826delC	p.P1609Qfs*41	Frameshift	55	<100	-	[18]

^1^ LRG_130t1.

**Table 2:** Combined criteria for the classification of novel APC variants based on the «Joint Consensus Recommendation of the American College of Medical Genetics and Genomics and the Association for Molecular Pathology» (2015) [17].

Nucleotide Change ^1^	Type of Variant	Criteria	Pathogenicity
c.1586_1587insAT	Frameshift	PVS1, PM1, PP1, PP3, PP4	Pathogenic
c.2336delT	Frameshift	PVS1, PM1, PP1, PP3, PP4	Pathogenic
c.3066_3067insGA	Frameshift	PVS1, PM1, PP1, PP3, PP4	Pathogenic
c.4303_4304insC	Frameshift	PVS, PM1, PP1, PP3, PP4	Pathogenic

^1^LRG_130t1. PVS1 – frameshift variant, PM1 – located in the critical and well-established functional domain, PP1 – co-segregation with the disease in multiple affected family members in a gene definitively known to cause the disease, PP3 – computational evidence support a deleterious effect on the gene product, PP4 – patient’s phenotype is highly specific for a disease.
